# Neurotrophins in carotid atherosclerosis and stenting

**DOI:** 10.1080/07853890.2022.2163052

**Published:** 2023-01-10

**Authors:** Teodora Yaneva-Sirakova, Latchezar Traykov, Kiril Karamfiloff, Ivo Petrov, Julieta Hristova, Dobrin Vassilev

**Affiliations:** a Acibadem City Clinic UMHAT, Sofia, Bulgaria; b Department of Neurology, UMHAT “Alexandrovska”, Neurology Clinic, Medical University Sofia, Sofia, Bulgaria; c Bulgarian Academy of Sciences, Sofia, Bulgaria; d Department of Internal medicine, UMHAT “Alexandrovska”, Cardiology Clinic, Medical University Sofia, Sofia, Bulgaria; e Department of Clinical Laboratory and Drug Toxicity, UMHAT “Alexandrovska” Clinical laboratory, Medical University Sofia, Sofia, Bulgaria; f Department of Health Care, UMHAT “Medica Cor”, Ruse, University of Ruse “Angel Kanchev”, Ruse, Bulgaria

**Keywords:** BDNF, NGF, carotid disease

## Abstract

**Introduction:**

Carotid stenting is used with an expanding indications. The neurotrophins are a family of proteins that induce the survival, development, and function of neurons. Carotid stenting alters cerebral blood flow and can affect neurotrophins’ levels.

**Material and methods:**

We included 78 people: 39 with significant carotid stenoses (CS) referred for carotid stenting (mean age 67.79 ± 10.53 years) and relatively healthy control group of 39 people without carotid and vertebral artery disease (mean age 57.42 ± 15.77 years). Brain derived reurotrophic factor (BDNF) and neuronal growth factor (NGF) concentrations were evaluated with ELISA method from venous blood – once for the control group; and for the carotid stenting group: before (n33), 24 h after (n22) and at least 1 month after (n18) carotid stenting.

**Results:**

There was a difference between the mean neurotrophins’ concentration of patients with significant carotid stenoses and the group without: BDNF *p* = 0.001, CI (-5.11 to −1.44) (3.10 ± 3.10 ng/ml in CS vs. 6.37 ± 4.67 ng/ml in controls); NGF *p* = 0.049, CI (0.64–347.75), 195.67 ± 495.34 pg/ml in CS vs. 21.48 ± 52.81 pg/ml in controls. BDNF levels before carotid stenting (3.10 ± 3.10 ng/ml) were significantly lower than the postprocedural (4.99 ± 2.57 ng/ml) – *p* < 0.0001, CI (-2.86 to −0.99). For NGF there was a tendency for lower values after stenting: 195.67 ± 495.34 pg/ml before vs. 94.92 ± 120.06 pg/ml after, but the result did not reach statistical significance. The neurotrophins levels one month after carotid stenting and controls’ were not significantly different *p* < 0.01 (BDNF 5.03 ± 4.75 ng/ml vs. 6.37 ± 4.67 ng/min; NGF 47.89 ± 54.68 pg/ml vs. 21.48 pg/ml).

**Discussion and conclusion:**

Periprocedural and mid-term concentrations of neurotrophins after carotid stenting change in non-linear model. This may be due to changes in cerebral perfusion and also might be involved in neuronal recovery and reparation after reperfusion.

**KEY MESSAGES**
- Periprocedural and mid-term concentrations of neurotrophins after carotid stenting change in non-linear model.
- As the majority of them are not specific, their periprocedural change can be used as a clinical correlate to guide changes or even success in carotid stenting.
- Changes in neutrophins’ concentrations may be due to changes in cerebral perfusion and also might be involved in neuronal recovery and reparation after reperfusion.
- This goes in analogy with cardiac high-sensitive troponin, used as procedural guidance in coronary interventions.

## Introduction

Neurotrophins are a group of biomarkers, which are involved in the development, plasticity, and function of central and peripheral nervous system [1]. Their function is mediated through two major receptor families: Trk (receptor tyrosine kinases) and p75NTR (TNF family) [2]. They are synthesized and secreted by sympathetic and sensory target organs. They are taken by the nerve endings via receptor-mediated endocytosis and transported retrogradely to promote reparation and differentiation [3]. Some neurotrophins can also be transported anterogradely and act on brain neurons [4]. Neurotrophins’ levels are dynamically changed depending on local/paracrine and systemic stimuli [5,6]. The most widely studied neurotrophins (in search of neurodegenerative treatment) are brain-derived neurotrophic factor (BDNF) and Neural growth factor (NGF). Both have specific role in neuronal functioning and reparation [5,7,8,9].

We hypothesize, that neurotrophins may also vary in response to acute and chronic hemodynamic changes resulting from carotid stenting. The aim of the current study was to evaluate the potential changes in the serum concentrations of BDNF and NGF in response to carotid stenting as compared to the mean values in relatively healthy controls without significant carotid artery stenoses.

Such a correlation between neurotrophins and carotid stenting it might provide a tool for monitoring of procedural outcome in endovascular treatment and explanation of expected brain/neuronal functional changes after the procedure. This should be viewed in analogy with the various biomarkers for coronary artery disease and outcomes in coronary stenting.

## Material and methods

We gathered full medical history, physical examination, and basic laboratory testing for all patients in the study. The major cardiovascular risk factors were defined with reference to the current guidelines for dyslipidaemia [10], thyroid disease [NICE Guidelines for thyroid dysfunction] [11] renal dysfunction[KDIGO guidelines for kidney dysfunction] [12] diabetes mellitus and impaired glucose tolerance [13,14] anaemia [15].

For the neurotrophins’ testing we used ELISA kit (Cusabio) – sandwich immune-sorbent method. Venous blood samples were taken and left to coagulate for 2 h at room temperature. Afterward – centrifugated and frozen at −70° C. The mean values of BDNF were given in ng/ml, and of NGF – in pg/ml. Blood samples were taken initially on inclusion in the study – BDNF pre/NGF pre; 24-hours after carotid stenting – BDNF post/NGF post; on follow-up at least one month later – BDNF follow-up/NGF follow-up. The control group had only one sampling – on inclusion of the study – BDNF control/NGF control.

Carotid ultrasound was done with Vivid E95 General Electric linear transducer 7–13 MHz with reference to the national and European recommendations. We used the NASCET method for assessment [16–19]. With reference to the cited recommendations, [20] we used the following definitions:

- Unstable plaque – with heterogenous structure with high risk for embolization.
- Stable plaque – homogenous structure, smooth surface, and good fibrous cap.
- Symptomatic patient – with transitory visual or neurological symptoms, non-specific symptoms which may be associated with brain ischemia.
- Non-significant plaque – less than 50% and without characteristics of unstable or high-risk, with peak systolic velocity less than 125 cm/sec and ratio Internal carotid artery/common carotid artery less than 2.5.

Echocardiography was done with Vivid E95 General Electric with reference to the European recommendations.

Carotid ultrasound, echocardiography and carotid angiography were performed as part of routine testing in the patients, who were referred for stenting. The control group was without angiographies as far as they were without indications for such.

Carotid angiography was done in the patients referred for stenting. All stenoses were assessed in at least two orthogonal planes. The quantitative analysis was done with software for angiographic analysis Dicom Works version 3.1.5.b, after proper calibration of the catheters in every case. Significant carotid stenos were those, which according to the NASCET method were at least 70%. We used carotid stenting as a method of treatment after a multidisciplinary discussion (‘Brain team’) between neurologist, cardiologist, angiologist and the leading interventionalist in carotid stenting in our center. The procedure was standard of treatment for the given patient, based entirely on medical grounds. The decision was based on the neurological symptoms, significance of the stenosis, atherosclerotic burden of the aortic arch, individual surgical risk. Those of the patients, who were not suitable for stenting, were referred for endarterectomy and were not included in this study. All the stenting procedures were done by a team of 2 interventionalists with cardiologic and angiology specialization, experience of more than 10 years in carotid stenting. Four (13%) of the patients were stented without distal protection, as it was impossible to introduce the spider through the tight stenosis. Carotid stenting was done on the basis of current guidelines and was preferred to endarterectomy in the cases that were suitable for stenting, as far as this procedure is gaining speed. There is also quite a lot of data on the endarterectomy, but the full potential of carotid stenting is still under investigation, especially when predictors of success are concerned.

Every patient was followed peri procedurally for neurological symptoms and hemodynamic parameters – blood pressure every 30 min and ECG monitoring.

SPSS 19 (IBM) was used for the statistical analysis, level of significance 0.05.

Inclusion criteria for the carotid stenosis group: Ejection fraction above 35% and significant carotid stenosis, referred for interventional treatment; without coronary and peripheral artery disease or fully revascularized before the procedure.

Exclusion criteria**:** acute cerebrovascular or cardiovascular incident at the time of hospitalization, acute heat failure, chronic heart failure with ejection fraction below 35%, acute renal or liver failure, atrial fibrillation with poor anticoagulation, severe anemia, acute major bleeding, Alzheimer’s disease, coma, inability to give informed consent, hemodialysis patients.

The control group consisted of relatively healthy and preserved people – without severe diffuse atherosclerosis of the carotid and other arteries; without carotid stenoses; with preserved flow and structure and without unresolved significant stenoses of the coronary or peripheral arteries, stable haemodynamics and complying with the general exclusion criteria. We did not administer any interventions in the control group, as far as they did not have indications for such.

**Figure 1.**
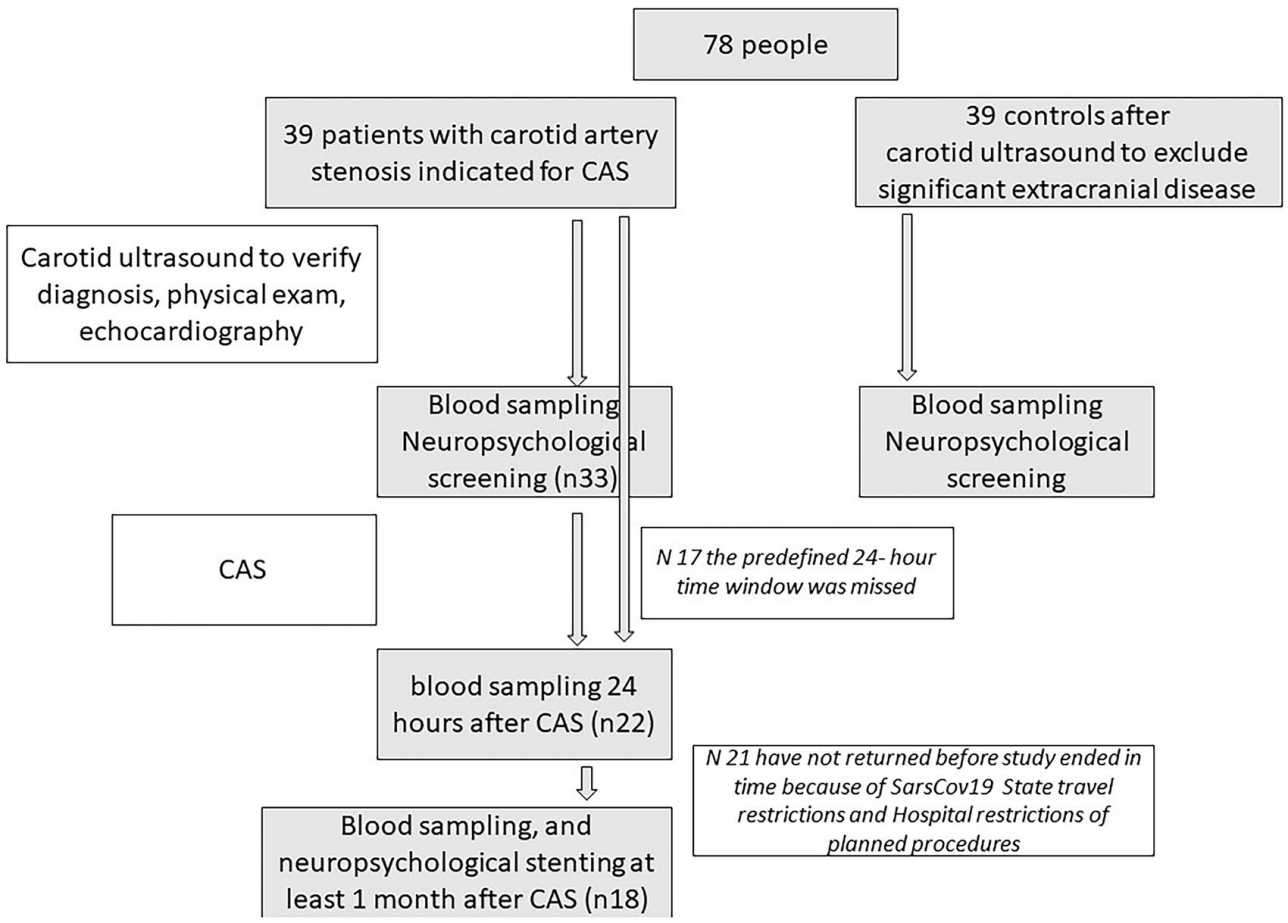
Study flowchart. Neuropsychological tests’ results are not discussed here. CAS: carotid stenting.

The flowchart of the study is given on Figure 1.

Ethical approval was granted by the Ethical Committee of Medical University Sofia, protocol 459/17.03.2020. All patients and controls signed written informed consent for participation of the study and their data personal and medical data were anonymized in accordance with the GDPR.

## Results

### Characteristics of the compared groups

We studied 39 consecutive patients with hemodynamically significant carotid stenoses, referred for carotid stenting. The control group consisted of 39 individuals without significant carotid artery disease.

*The group with significant carotid stenoses:* mean age was 67.79 ± 10.53 years; the males were 28 (72%) and 11 (28%) the females. The ratio males/females corresponded to the specific one for the general population for extracranial atherosclerotic disease [21]. Twenty-three (59%) were with previous stroke or transitory insufficiency of the brain perfusion 5(13%); in 10 (27%) the stenoses were unilateral, with only non-significant mild stenoses in the contralateral internal carotid artery; significant bilateral stenoses had 12 (32%); contralateral occlusion had 7 (18%); 5 (13%) of the revascularized patients were with severe stenoses and one bilaterally [22].

Interventional characteristics: 16 (50%) of the stented patients experience periprocedural hypotension, reversed with volume infusion and vasopressor if hypotension persisted. Distal protection was not used only in 4 (13%) of the patients. The stent types used were: Wallstent 17(53%); Cristalo ideale 1(3%); Protégé RX 12(37.5%); Precise Pro RX 1(3%); balloon dilation 1(3%). The percent is more than 100%, because in several isolated cases we had more than 1 stent implanted.

*Control group:* the mean age was 57.59 ± 15.60 years, 15(38%) were males, only 3 (7.7%) had previous stroke more than 6 months before the index inclusion in the study.

The two groups – indicated for stenting and without significant carotid stenoses were compared with *T* test. We found significant difference in age (*p* = 0.001, CI 4.2–16.21 the mean age in the group with carotid stenting was 67.79 ± 10.53 years, controls −57.59 ± 15.60 years); sex [*p* = 0.03, CI 0.12–0.55, the males in the stented group were 28 (72%), in the control group − 15(38%)]; previous stroke (*p* < 0.0001, CI 0.33–0.69, 23(59%) in the stented group and 3 (7.7%) in the control group). The comparison with reference to the major cardio-vascular risk factors and diseases was given on Table 1.

**Table 1.** Comparison between the mean values or frequencies of cardio-vascular risk factors and diseases in patients referred for stenting and the control group without significant carotid artery disease.

Variable	With CAS	Controls	*р*	CI
Arterial hypertension, (*n*, %)	38 (97%)	27 (69%)	0.001	0.12–0.44
Systolic blood pressure, mmHg	136.53 ± 14.56	122.43 ± 13.80	<0.0001	7.7–20.50
Diastolic blood pressure, mmHg	80.12 ± 7.56	74.61 ± 10.22	0.009	1.45–9.57
Pusle pressure, mmHg	56.41 ± 11.75	47.82 ± 6.26	<0.0001	4.32–12.86
Mean pulse pressure, mmHg	97.28 ± 8.71	89.45 ± 10.56	0.001	3.46–12.19
Hypouricemia, (*n*, %)	5 (13%)	4 (10%)	0.73	–0.12–0.17
Dyslipidemia, (*n*, %)	30 (77%)	25 (64%)	0.22	–0.08–0.33
Obesity, (*n*, %)	8 (20%)	5 (13%)	0.37	–0.09–0.25
Diabetes mellitus, (*n*, %)	16 (41%)	11 (28%)	0.24	–0.09–0.34
Mild to moderate anemia, (*n*, %)	8 (20%)	2 (5%)	0.04	0.004–0.30
Renal dysfunction, (*n*, %)	5 (13%)	4 (10%)	0.73	–0.12–0.17
Creatinine, (mkmol/l ± SD)	87.38 ± 33.06	87.10 ± 19.30	0.97	–13.29–13.88
Compensated thyroid dysfunction, (*n*, %)	6 (15%)	8 (21%)	0.06	–0.27–0.16
Atrial fibrillation, (*n*, %)	10 (26%)	7 (18%)	0.42	–0.11–0.26
Previous STEMI, (*n*, %)	2 (5%)	8 (20%)	0.04	–0.30– −0.005
Coronary artery disease, (*n*, %)	27 (69%)	15 (38%)	0.006	0.09–0.52
Peripheral artery disease, (*n,* %)	12 (31%)	2 (5%)	0.003	0.09–0.42
Renal artery stenosis, (*n*, %)	4 (10%)	1 (2%)	0.25	–0.05–0.21
Diastolic dysfunction, (*n*, %)	33 (84%)	29 (74%)	0.27	–0.08–0.28
Ejection fraction, (*n*, %)	59.18 ± 8.97	56.51 ± 11.41	0.25	–1.96–7.29
EDV (ml)	109.11 ± 36.00	119.63 ± 37.14	0.24	–28.44–7.47
ESV (ml)	45.05 ± 19.92	56.27 ± 28.10	0.06	–23.04–0.59
Chronic obstructive pulmonary disease, (*n*, %)	4 (10%)	5 (13%)	0.73	–0.17–0.12
Smoking, (*n*, %)	21 (54%)	18 (46%)	0.50	–0.15–0.30
Family history for early atherosclerosis, (*n,* %)	7 (18%)	10 (26%)	0.42	–0.26–0.11

CAS: carotid stenting.

Blood pressure measurements in the table were the reported mean home measured values form the previous week.

### Results with the neurotrophins’ concentrations

On Table 2, we showed the mean values of BDNF and NGF depending on the sampling period. The values of BDNF were in ng/ml, of NGF – in pg/ml.

**Table 2.** Mean values of BDNF and NGF in the main groups and in the different sampling periods.

	BDNF before CAS	BDNF After CAS	BDNF follow-up	BDNF controls	NGF Before CAS	NGF After CAS	NGF follow-up	NGF controls
Mean ± SD	3.10	4.99	5.03	6.37	195.67	94.92	47.89	21.48
	3.10	2.57	4.75	4.67	495.34	120.06	54.68	52.81
*n*	34	22	18	39	34	22	18	39

CAS: carotid stenting.

**Figure 2.**
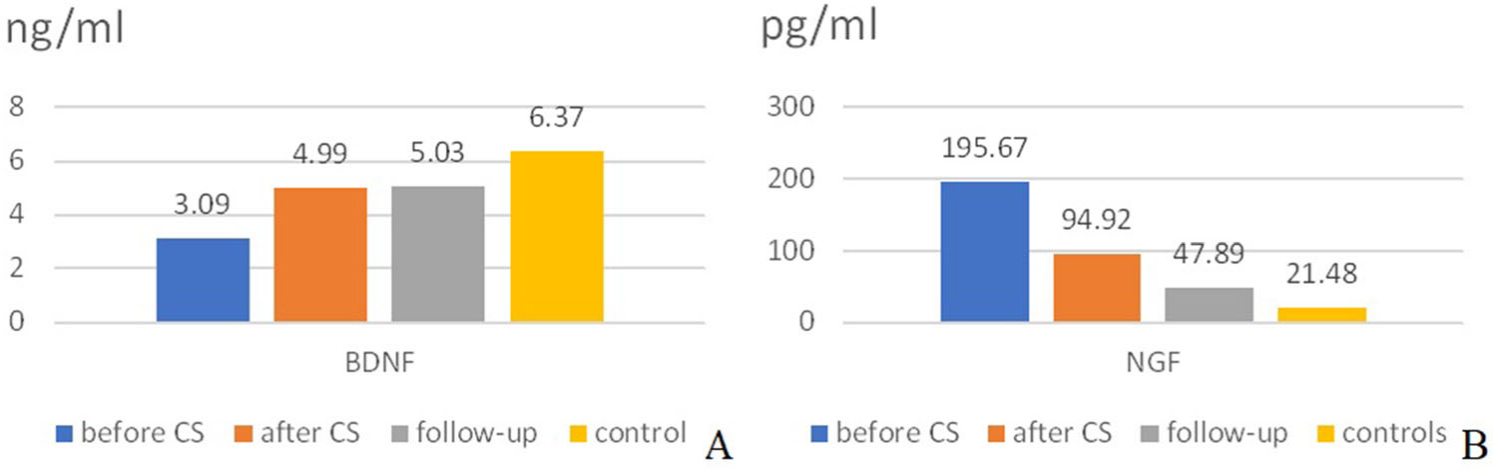
Mean concentration of neurotrophins depending on the sampling time with reference to CAS or in the control group. (A) BDNF; (B) NGF.

The temporal dynamics of BDNF and NGF concentration was given on Figure 2.

The mean values of BDNF generally rose after stenting, and the mean values of NGF generally fell after stenting. However, statistically different were only the values of BDNF before (3.10 ± 3.10 ng/ml) and after CAS (4.99 ± 2.57 ng/ml), *p* < 0.0001, CI (-2.86 to −0.99 paired sample *T* test). There was difference between the other corresponding pairs, but it did not reach statistical significance.

Independent samples *T* test was used to compare the mean values of BDNF (respectively NGF) between the group with CAS before CAS and the control group. The mean values and standrd deviation were shown on Table 2. There was a significant difference in the mean values: BDNF was 3.10 ± 3.10 ng/ml for those with significant stenoses before CAS and 6.37 ± 4.67 ng/ml for the control group [*p* = 0.001, CI (-5.11 to −1.44)]; NGF was *p* (195.67 ± 495.34 pg/ml for patients with significant stenoses before CAS and 21.48 ± 52.81 pg/ml) [*p*=0.049, CI (0.64–347.75)].

The difference between the mean values of the neurotrophins on follow-up after CAS and the control group was not significant. There was a tendency for lower values in the control group (*p* = 0.065), however it did not reach statistical significance.

None of our patients had neurological complications after stenting.

There was no correlation between age or gender or coronary artery disease and neurotrophins’ levels in our study. The regression analysis showed also associations of neurotrophins with systolic and pulse pressure, but this was not the topic of the current paper.

## Discussion

There are several major findings from the above study, which may be of importance for the future research and the search of biomarkers to guide carotid interventions.

*There was a significant difference in the mean values of BDNF and NGF between the groups with significant carotid stenoses referred for stenting, and those without significant carotid stenoses (‘significant’ was defined in Methods part).* Several studies in the field were with reference to this finding. Li et al. [23] found that chronic brain hypoperfusion and Aβ leaded to downregulation of gene expression for BDNF mRNA. In a study in rats [24] with induced cerebral ischemia, NGF secretion in the cortex showed temporal and loco-specific profile – absent in the infarcted area, with retarded compensatory elevation in the penumbra area and higher midterm values in the healthy regions, probably stimulating the reparation in the affected neurons [25]. Another study of Lindvall et al. [26] proved temporal response of NGF mRNA to acute cerebral ischemia.

*The mean values of neurotrophins in patients with carotid stenting varied dynamically throughout the studied period – before, 24 h after stenting and after at least one month follow-up.* Our search of the literature couldn’t find data on the possible significance of neurotrophins in carotid stenting. These results were preliminary on the topic. We saw a specific pattern peri procedurally, however the result did not reach statistical significance and thus, it needs further exploration with neuroimaging, as we did not have enough neurological complications to base any clinically meaningful result on this outcome.

*The mean values of BDNF after carotid stenting were significantly higher than the mean values before. This result should be correlated with fMRI in other to confirm whether BDNF could be used in periprocedural monitoring and assessment of functional neurocognitive outcome in carotid stenting.* The result went in line with the discussed studies for lower BDNF levels in atherosclerosis and endothelial dysfunction, and low levels in cerebral hypoperfusion [24]. With stenting the cerebral perfusion was improved [25]. On the other hand, in a study of stroke patients Bejot et al. [26] found that the acute phase circulating BDNF concentrations did not mirror brain BDNF and severe stroke was correlated with high plasma BDNF concentrations – BDNF rise in concentration was as a response to high neuronal demand activation of reparative processes. In our study, distal embolic protection devices were used in nearly all our patients; however, we did not use periprocedural transcranial Doppler embolic monitoring. Despite the lack of severe postprocedure neurological symptoms, we did not have objective evidence for the quality of the embolic protection and thus, there may be several conflicting explanations for the higher postprocedural BDNF – either better perfusion or higher demand. However, we consider the result important in the light of future thorough explanation of the role of biomarkers in interventional procedures for carotid stenoses [27,28].

## Conclusion

There may be some association between significant carotid disease and neurotrophins’ serum levels. Periprocedural and mid-term concentrations of neurotrophins change in non-linear model. This may be due to the improved perfusion and altered brain hemodynamics, as well as to the involvement of neurotrophins in the neuronal recovery and reparation. These are the preliminary results for a possible future procedural monitoring and practical use of neurotrophins.

## Limitations

The study is relatively small, but we hope it will be the foundation for further research in the field – the significance of peripheral neurotrophins’ levels as diagnostic and prognostic markers in cerebrovascular diseases. We do not have a control group of patients with significant carotid stenosis on optimal medical therapy. This was due on the inclusion criteria based on the current guidelines for indications for revascularization. A correlation with neuroimaging is needed. As this was a clinical study, an animal model should be used to prove our hypothesis.

## Clinical significance

We have biochemical biomarkers, which guide coronary interventions and are used for prognostic assessment. We are in need of similar biochemical biomarkers in carotid interventions, periprocedural success and long-term assessment in patients with carotid artery disease. This is a study for the possible incorporation of neurotrophins in the periprocedural assessment of carotid stenosis and stenting.

## Data Availability

Data available on reasonable request from the corresponding author.
